# Remarks on Blood-Lettting

**Published:** 1847-01

**Authors:** 


### ART. II.—*Remarks on Blood-letting.* By the Editor.

[Concluded from Page 388.]

I pass next to speak of blood-letting in fever. Here, as with reference to inflammation, it is necessary to remark, that regard for the brevity due to this occasion will preclude so full a discussion as would be required if we were not restricted to the limits of a single discourse. Let me inquire, in the first place, what is the testimony of experience concerning blood-letting in fevers? For if there be a positive answer to this inquiry, and a satisfactory unanimity among medical men, this fact would go far toward placing the subject upon safe and incontrovertable ground. It must, however, be confessed that, as in the former case, we receive from this source a diversity of doctrine. Individual opinions of those enjoying extensive opportunities for observation, possessing ample mental qualifications and industry to improve their opportunities, and whose honesty of intention is unquestionable, have always differed widely, and still differ on this point. This, of itself, leads to some important presumptions. It is fair to infer from this discrepancy:

1st. That blood-letting may be appropriate in fever, and may be carried to considerable extent without notable injury.

2d. That it is not always necessary, and may be injurious.

3d. That the disease as it is presented to the observation of different practitioners, in different places, seasons and periods, is widely different as respects its tolerance of this remedy.

This last inference reconciles much of the discrepancy which exists between the two first, and sufficiently explains why the opinions of different observers of equal sagacity, founded on their own experience, are so opposite.

The application to the treatment of fever of the same general principles which have been presented in our remarks on inflammation, taken in connexion with the general views entertained on the subject by the most eminent pathologists, will, if I mistake not, exhibit a coincidence which seem to confirm their correctness. Let me briefly repeat them. Blood-letting has been considered as adapted to obviate a condition in which there is an actual increase of the mass in circulation beyond its proportionate relation to the solids; and to a condition in effect identical, arising from an interruption of the usual expenditure in the processes of appropriation and elimination, conjoined with an increased rapidity of the movement of the blood. In general, we limited the extent of its application to the removal of this condition; but we considered several modifications (omitting others) of the general rule, derived from the differences of tissue affected, the peculiar relations of certain organs to the circulation, and the co-operation of peculiar and inappreciable morbid agencies. Now we have in fever frequently the same condition. General hyperæmia may have existed previous to, and at the commencement of the attack, as in the case of inflammations; and, perhaps, as in the latter case, contribute to its devolopment. Is it not probable that this will serve, as in the case of inflammations, to explain the few instances in which fever appears to be cut short in its incipient stages? Blood-letting, therefore, in such cases will be useful, and if the inquiry just made be answered affirmatively, may be resorted to under these circumstances with some confidence of preventing the farther progress of the disease.

When this condition of the circulation does not precede or accompany fever, it is often developed by the morbid actions which characterise the febrile movement. The normal activity of the secretions, of nutrition, and of the exhalations, are at once interrupted; hence the portion which by the renewal of the tissues, and the different emunctories, is constantly being withdrawn from the mass, is now in a great measure retained in the circulation; and, in addition to this, the increased action of the central organ causes the blood to perform its two circuits with greater rapidity. This will account for that condition which we ordinarily call inflammatory, or, to use the expression of Fordyce, "general inflammation," which characterises some fevers at their commencement, as well as those inflammations which are distinguished as *active*. As this general condition in inflammation reacts upon the local disease, augmenting its intensity, so we have every reason to believe, theoretically, and not less from practical observation, that it disposes to the local lesions, which we know generally complicate febrile affections, and in which the danger chiefly consists. For precisely the same reasons, therefore, in the latter case as in the former, bleeding is a most important, and, it may be, *the* most important remedy. The only modifying consideration which in this application occurs to me as important in a general view is, that the dangers from an abuse of the remedy are greater, inasmuch as the probabilities are that a febrile affection will have a longer duration, before the processes of restoration commence, than an acute inflammation, and that the evils which may be induced by an excessive abstraction of blood should lead us to exercise even more caution and discrimination, lest, in our efforts to remove simply an exagerated condition of the circulation, we sacrifice an indispensable element of recuperative energy.

It is superfluous to add, that the same remarks which were made respecting the application of this remedy to certain local inflammations—of the heart, lungs and brain—are equally pertinent when these are presented as febrile complications.

But do these principles apply in the same measure to all fevers? Far otherwise. It will be recollected while speaking of inflammations we noticed briefly the fact that exceptions occur from the influence of general and inappreciable agencies, operating frequently on masses of individuals simultaneously, and sometimes in single cases, giving to the disease a specific character, and that in our ignorance of the causes and consequent pathological modifications, in the present state of the science, it is impossible to bring these cases within the sphere of rational and general principles of therapeutics. This is true, and to a greater extent, in febrile diseases. The greater part of those cases embraced within the nosological class called typhus, and, in general, those which we are in the habit of characterizing as typhoid, adynamic, ataxic, putrid, &c. stand in the same relation to this and other remedies of the depletory and antiphlogistic class, as do cases of inflammation modified by epidemic, endemic and other influences communicating to them an asthenic character. As before remarked, it would be interesting to inquire concerning the nature of these modifying agencies, and the mechanism of their operation, but no apology is necessary for waiving it on this occasion. I would simply remark, that in both inflammations and fevers of this character, the action of the modifying causes seems to be manifested more especially in the circulation, inducing a condition different from that which determines us to use the lancet, and assists us to regulate judiciously the extent of its remedial adaptation.

I had designed to have considered blood-letting in its relations to other forms of disease, especially congestion and hemorrhage, but the space which I have already occupied admonishes me to bring my remarks to a close. In pursuing the subject in its connection with the diseases just mentioned, the same general principles would be found susceptible of application, with exceptional modifications of an important character. It is an interesting, and, in a practical view, exceedingly important fact, that these diseases arise under directly opposite causative conditions; the one demanding blood-letting as the most effective remedial agent, and the other requiring not less imperatively its forbearance. The vital consequences to the patient of a proper discrimination is too obvious to require notice.

The general doctrine which I have maintained will probably appear to some who hear me to limit too much the efficacy of blood-letting, and to exhibit the dangers of its abuse in too prominent a point of view. I am fully aware that the views I have been led to adopt are not in accordance with those entertained by many writers and practitioners, who are entitled to all the respect which authority has a right to claim from the members of a profession, imposing great responsibility upon the formation of opinions which are to affect the health and lives of our fellow beings. So much diversity, however, has always existed, and still exist, among the profession in the degree of confidence in this remedy, that no peculiar doctrine can properly expose any member of the profession, however humble his pretensions may be, to the charge of undue presumption. We are bound in all such topics to take into consideration the conclusions to which others have arrived, and to concentrate upon them all the light which may be reflected by new developments constantly transpiring in the progress of science. If I regard the present aspect of pathology aright, the remedial agent we have treated of is assuming a position somewhat different from that it has heretofore occupied—a position intermediate between the two extremes of abuse and neglect; between too much and too little confidence. In other words its efficacy is of late becoming to consist more emphatically in a nice discrimination of the pathological conditions to which it is applicable. Several improvements in pathology and diagnosis have contributed to this end. Among these may be mentioned as prominent, the elucidation of anæmia, and the various phenomena dependent on this state. Who that has observed diseases before and after appreciating this pathological condition, can fail to be convinced from his own experience, that the indiscriminate employment of blood-letting in fevers, inflammations, hemorrhagies, &c., has been injurious in its consequences to an extent which it is melancholy to think of!

The enlarged sphere which the neuroses occupy in recent pathology, is another prominent fact, which has affected our estimation of this therapeupic agent. This increased space has been acquired at the expense of the class of supposed inflammatory affections. One who has had but a few year's experience in the profession, has been able to perceive a great change in this particular. It is but lately that neurotic affections extended but little beyond the *tic doloreux;* now, how large a proportion of cases in which pain is the predominant symptom, are known by the appellation of neuralgia! There are probably none of us who cannot remember how frequently bleedings and depletory remedies were formerly employed for affections which now we should not hesitate to pronounce neuralgic. Modern pathology reveals to us the fact that neuralgic affections are among the common effects of the anemic state; we can, therefore, readily conceive what unfortunate results have followed errors of diagnosis unavoidably incident to the prevailing imperfections of science. Our knowledge of the essential cause of inflammation, although it cannot be said to have advanced to any positive discoveries, is, nevertheless, nearer the truth, in as much as it is divested of the erroneous hypothses which formerly prevailed. The influences which these have exerted in affecting the estimation of blood-letting I have already alluded to. Another evidence of progress, affecting this, in common with other remedies, is the recognition of the fact, that most diseases involve in themselves a certain career of continuance and termination, in other words, that they are self-limited. Other considerations might also be mentioned in the same category.

Finally, a better appreciation of what has been called medical experience, and a conviction of what science really requires in order that practi·al observation and experimental research should constitute reliable evidence, render the physician at this time more cautious in admitting principles of therapeutics, especially when they involve ultraism, either of faith or skepticism. It is not to be contended that the old maxim '*in medio tutissimus ibis*,' possesses any abstract virtue as a guide to the formation of medical opinions, yet it is fair to infer from the discrepancy exhibited by practical observers, and sound thinkers on this, as well as numerous other topics in medicine, that truth lies somewhere between the two extremes.

Let me not be thought by these remarks to disparage *experience* in medicine. It is the last and highest tribunal to which the mind in its inquiries for medical truth can appeal. Although it may reverse the decisions arrived at by logical deductions which appeared unimpeachable, its authority is supreme, and cannot be questioned. But true scientific experience is something widely different from what has heretofore generally passed under this title. It is not made up of facts carelessly observed, collected at long intervals, and recalled afterward by an effort of recollection; occurring in the limited sphere of single individuals, acting without concert, each too often regarding every thing through the medium of preconceived doctrines. Medical experience has hitherto too much resembled the mode in which some fanatics study the writings of inspiration, to establish, by detaching disconnected passages, a peculiar creed of their own, or the doctrines to which they have committed themselves without examination. Thanks to the progressive character of our science—experience has now a different signification! And I have full confidence to believe that the time will come, when, by the harmonious combination of rational and experimental inductions, principles of therapeutics will be established on bases, which, compared with their present foundations, will be permanent and immoveable.

